# Identification of the soil physicochemical and bacterial indicators for soil organic carbon and nitrogen transformation under the wheat straw returning

**DOI:** 10.1371/journal.pone.0299054

**Published:** 2024-04-04

**Authors:** Yajun Yang, Hui Wang, Chengjuan Li, Hexiang Liu, Xianhui Fang, Mengyuan Wu, Jialong Lv

**Affiliations:** 1 State Key Laboratory of Soil Erosion and Dryland Farming on the Loess Plateau, Institute of soil and water conservation Chinese Academy of Sciences & College of Natural Resources and Environment, Northwest A&F University, Yangling, Shaanxi Province, PR China; 2 Xianyang Soil and Fertilizer Workstation, Xianyang, Shaanxi Province, PR China; Tennessee State University, UNITED STATES

## Abstract

Wheat straw returning is widely practiced in agriculture; therefore, it is critical to determine the physicochemical and bacterial indicators in soil for the organic carbon storage, accumulative C mineralization, total nitrogen improvement, and nitrogen mineralization in various soil types after wheat straw returning. This study evaluated the influenced indicators of wheat straw addition on soil organic carbon and nitrogen transformation in diverse soil types. For this purpose, an incubation experiment was conducted to analyze the carbon and nitrogen transformation in soil from eight Chinese provinces treated with the same dry weight of wheat straw. The results indicated that the primary physicochemical and bacterial indicators that predict the carbon and nitrogen transformations in the acidic and alkaline soils were different. Of all the natural physicochemical properties of soil, cation exchange capacity and clay content were significantly correlated with organic carbon, mineralized carbon, total nitrogen, and mineralized nitrogen in the alkaline soil. In the acidic soil, the initial C/N ratio of soil was the most significant indicator of carbon and nitrogen transformation. From the perspective of the carbon- and nitrogen-relating bacterial communities, *Proteobacteria* were largely responsible for the accumulative C mineralization in both types of soil. Furthermore, *Proteobacteria* strongly regulated the organic carbon storage in the acidic soil after wheat straw addition, whereas *Gemmatimonadetes* was the main predicted indicator in the alkaline soil. Additionally, total nitrogen and mineralized nitrogen levels were largely explained by *Bifidobacterium* and *Luteimonas* in the alkaline soil and by *Nitrospira* and *Bdellovibrio* in the acidic soil. Soil physicochemical and biological properties significantly influence soil carbon and nitrogen transformation, which should be considered crucial indicators to guide the rational regulation of straw return in several areas.

## 1. Introduction

With the rapid development of agricultural production in China, a substantial increase in wheat straw generation has been observed. Wheat straw production reached 980 million tons in China, but the comprehensive utilization rate of straw was only 81.68% [1–3]. Effective wheat straw management is of social and environmental concern. There are several methods to dispose wheat straw, and wheat straw returning is the most widely adopted approach in most countries [4]. Straw, as an important renewable resource, contains mineral nutrients and a large amount of organic matter (OM) required for plant growth. Straw returning proves can introduce new OM to the soil [5], making it an effective method for enhancing soil organic carbon (OC) and total nitrogen (TN) content and for promoting soil fertility [6–9].

After returning, wheat straw undergoes mineralization and humification through a series of biochemical reactions [10]. OM in the wheat straw comprises biodegradable and recalcitrant components. Recalcitrant component is not sensitive to short-term changes in soil management, whereas biodegradable component is sensitive to soil management [11, 12]. Mineralization refers to the conversion of complex biodegradable components in wheat straw to simple components through the action of microorganisms [13]. Carbon dioxide (CO_2_) emission during mineralization has been proven to have a significant impact on the global carbon cycle and climate change [14, 15]. Recalcitrant components contribute more to carbon accumulation than biodegradable components [16, 17]. Furthermore, humification refers to microorganisms that use intermediate products that are produced after wheat straw decomposition to generate synthetic products and metabolites, which are then partly mineralized, partly converted into soil OC and nitrogen, and finally condensed into humus components [18]. Humus was considered the most stable portion of soil OM [19]. Previous studies have reported that wheat straw returning significantly increased soil OC sequestration [6, 20, 21] and nitrogen level [22, 23] in individual or multiple soils [24, 25]. Additionally, extensive research has revealed that the soil OC transformation differed with the soil type due to environmental variables [26]. However, the decomposition and accumulation of soil OC and TN in multiple soil types after straw returning remain unclear. Therefore, the carbon and nitrogen transformation after wheat straw returning in several soil types requires further research.

Soil microorganisms are an indispensable component of agricultural ecosystems [27]. They are almost involved in all key processes of agricultural production, and soil biological indicators could respond rapidly to changes in soil conditions [28]. Microbes play a significant role in the decomposition and accumulation of soil carbon and nitrogen. Guo et al. [29] also reported that the application of organic amendments altered the distribution of microbial communities. Zhao et al. [25] discovered that high doses of maize straw application modified the microbial community structure. Garcia-Pausas and Paterson [30] observed that OC mineralization significantly correlated with the abundance and structure of bacterial communities. Diverse active microorganisms metabolized in soil due to the complexity of the soil environment [31]. Therefore, proper management of soil microbial communities is of great significance in sustainable agricultural ecosystems [32]. Several studies revealed that various microorganisms play different roles in soil OC and TN mineralization and stabilization. The distribution of microorganisms in different soil types may differ due to the soil properties, which then affects the transformation of soil OC and TN content in various soil types. However, the impact of straw returning to the field on soil microbial communities and its indicative role in soil OC and TN conversion are still unclear.

Although straw returning to the field is a common agricultural production measure in China. However, there are significant differences in planting systems, climate conditions, soil conditions, straw types and application rates under actual field conditions, making it difficult to accurately explain the microbial driven effects of straw returning on soil OC and TN transformation [6]. The incubation experiment provides a better opportunity to solve this scientific issue. Therefore, this study investigated the succession of microbial communities and the transformation of soil OC and TN in several soil types after wheat straw addition. This study aimed to reveal the OC and TN decomposition and transformation in different soil types and identify its physicochemical and microbial factors.

## 2. Materials and methods

### 2.1. Soil, materials and experimental setup

Soils for the incubation experiment were collected from the surface (0–20 cm deep) in eight provinces, including Yunnan (YN), Jiangxi (JX), Gansu (GS), Jilin (JL), Henan (HN), Inner Mongolia (IM), Tianjin (TJ), and Chongqing (CQ), which are primary wheat-producing areas. Since the samples were obtained from farms, not experimental fields, no permits were required to conduct this work. The soil samples were air-dried, sieved, and thoroughly mixed with 5% wheat straw in the laboratory. The wheat straw was collected after harvesting crops from farms in Yangling, Shaanxi Province, China. The straw was then cut into < 2 mm pieces for incubation. The properties of wheat straw were as follows: OC of 406.6 ± 2.13 g kg^-1^, TN of 8.06 ± 0.14 g kg^-1^, C/N of 50.42, pH of 6.52 ± 0.05.

The incubation experiment in triplicates were conducted at 25°C in the dark for 77 days. For each treatment, 500 g soil was thoroughly mixed with the same amount of wheat straw (5%, w/w dry basis) and incubated in a 1000 ml plastic cup. Every three days, deionized water was added into cups to maintain 70% moisture holding capacity. Soil samples were collected on days 0 and 77 of incubation. Air circulation was ensured by opening the cups on each test day. A small bottle containing 20 ml sodium hydroxide (NaOH, 0.5 mol/L) was placed in each cup for CO_2_ absorption and removed on days 1, 2, 3, 4, 5, 6, 7, 11, 14, 21, 27, 49, and 77 of the incubation.

### 2.2. Determination methods

CO_2_ emission was determined by titrating with 0.5 mol/L HCl and calculated as follows:

$${CO}_{2}-C=\frac{(V_{0}-V)\times C_{Hcl}}{2}\times 44\times \frac{12}{44}\times \frac{1}{m\times (1-a\%)}$$

where CO_2_-C is the amount of mineralized soil OC (g/kg); V is the consumed volume of titrated HCl (mL); V0 is the standard consumed volume of HCl (mL); C_HCl_ is HCl concentration (mol/L); a% refers to the soil moisture content and m means soil weight for testing.

At each designated sampling time, soil OC was measured by oxidation with potassium dichromate and titration with ferrous ammonium sulfate. The total nitrogen content was determined using the Kjeldahl method. Further, pH was measured using a pH meter at a soil:water ratio of 1:2.5. Ammonium acetate was used for measuring the cation exchange capacity (CEC) at pH 7.0 [33]. Clay content was determined using the standard pipet method, and calcium carbonate (CaCO_3_) concentration was measured using the pneumatic method. Available potassium (AK) content was measured via flame photometry after ammonium acetate extraction. Available phosphorus (AP) content was extracted with a 0.5 M NaHCO_3_ solution under pH of 8.5 and measured using a spectrophotometer [34]. The selected soil properties are shown in S1 Table. On day 77, a portion of the soil samples was collected and stored at—20°C for 16S rDNA sequencing. After the extraction, assessment, and quantification of DNA samples, the 16S rDNA sequencing was amplified in the V3-V4 region with the primers 338F and 806R and determined on the IlluminaMiSeq PE300 sequencing platform. The Miseq sequencing obtained Pair-End double ended sequence data, and the fastq data was subjected to quality control processing, ultimately resulting in high-quality fasta data. The fasta data was stored in a database SRA (Sequence Read Archive, http://www.ncbi.nlm.nih.gov/Traces/sra) established by the NCBI data center. Operational taxonomic units (OTUs) were clustered with a 97% similarity cutoff using UPARSE version 7.1 (http://drive5.com/uparse/), and chimeric sequences were identified and removed using UCHIME. The phylogenetic affiliation of each 16S rDNA gene sequence was analyzed by the UCLUST algorithm. The functional carbon-relating and nitrogen-relating bacteria were screened according to the previous researches [35–37]. The related functional genes were predicted by the PICRUSt (Phylogenetic Investigation of Communities by Reconstruction of Unobserved States).

### 2.3. Data analysis

Figures were prepared using Origin 2016. The Spearman correlation analysis, significant differences under Duncan’s multiple range test, and linear regression analysis were performed by IBM SPSS 23.0 version. Heat map analysis was conducted using Heml. The explanation of physicochemical and biological indicators for carbon and nitrogen transformations was calculated based on the redundancy analysis (RDA) including 3 replicates, which was conducted by CANOCO 5.0.

## 3. Results

### 3.1. Accumulative CO_2_-C mineralization and soil OC

**Fig 1:**
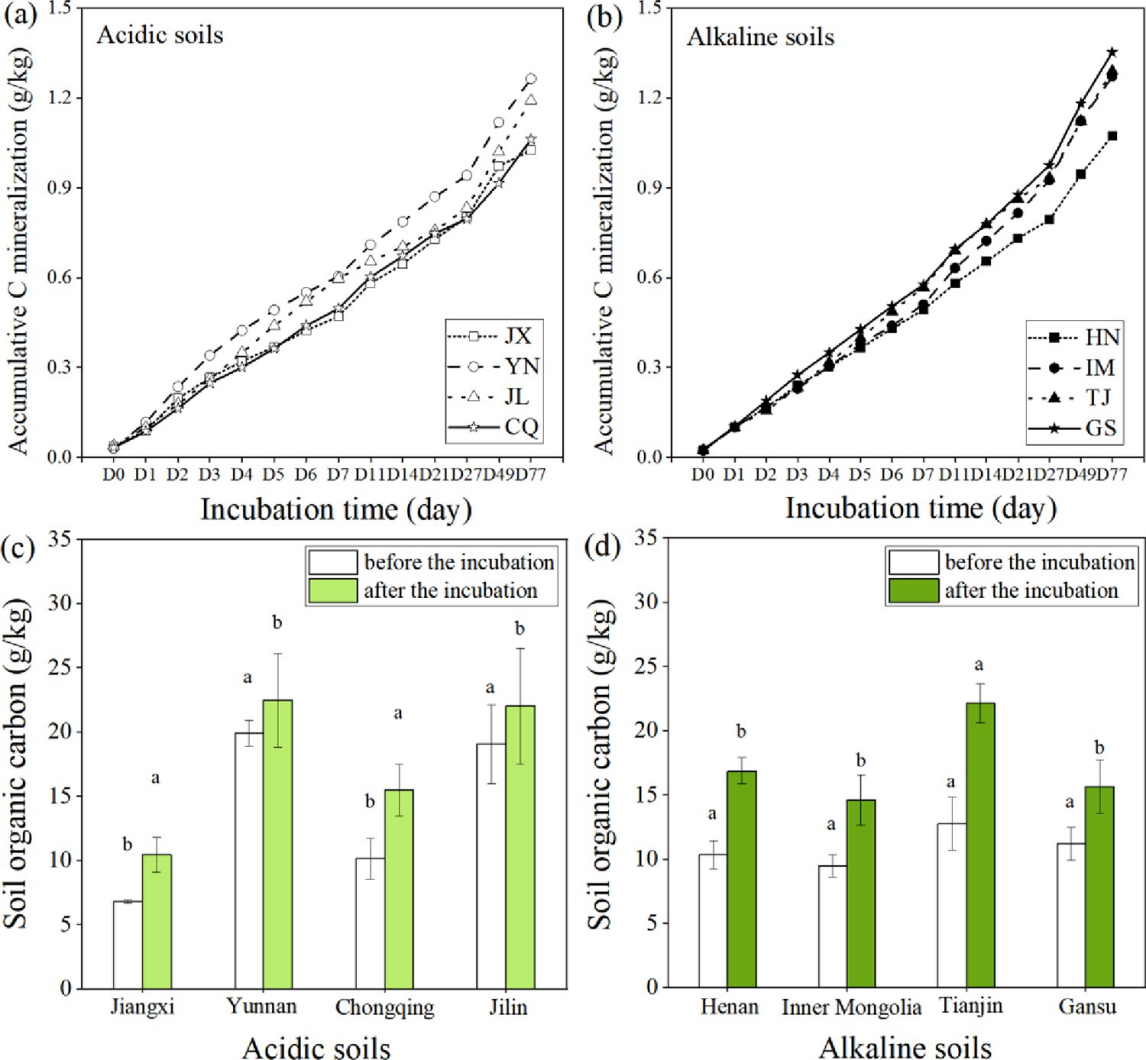
Accumulative C mineralizations after the incubation in the acidic (a) and alkaline soils (b); soil organic carbon content before and after the incubation in acidic (c) and alkaline soils (d) (Values are the mean (n = 3). Different lowercase letters within a column indicate significant differences at the 5% level. Significant difference was performed between that in different types of soils before and after the incubation; JX: Jiangxi; JL: Jilin; YN: Yunnan; CQ: Chongqing; GS: Gansu; IM: Inner Mongolia; TJ: Tianjin; HN: Henan).

Accumulative CO_2_-C mineralizations exhibited a similar increasing trend in different soil types after wheat straw addition (Fig 1). In the acidic soil, the accumulative CO_2_-C mineralization was in the range of 1.03 g/kg—1.26 g/kg, and the maximum value was observed in the YN sample, followed by those in JL, CQ, and JX soil samples (Fig 1A). Compared with that in the YN sample, the accumulative CO_2_-C mineralization significantly decreased by 5.71%, 15.92%, and 18.88% in the JL, CQ and JX soils, respectively (*P* < 0.05); however, there was no significant difference in the emission in CQ and JX soils after wheat straw addition (*P* > 0.05). In the alkaline soil, accumulative CO_2_-C mineralization in the GS, TJ, IM and HN soils ranged from 1.07 g/kg to 1.35 g/kg; the accumulative CO_2_-C mineralization in the GS sample was 25.96%, 6.37%, and 4.87% higher than those in the HN, IM and TJ soil samples, respectively (Fig 1B). There was a significant difference in the cumulative CO_2_-C mineralization of HN and other soil samples (*P* < 0.05), whereas the differences among those of the IM, TJ, and GS samples were insignificant (*P* > 0.05).

The soil OC content in the various soil types before and after wheat straw addition were shown in Fig 1. Wheat straw addition significantly increased the soil OC content in the acidic and alkaline soils (*P* < 0.05) from 13.09% to 53.78% and from 39.89% to 73.22%, respectively. In the acidic soil, the OC was in the range of 10.43 g/kg—22.47 g/kg after wheat straw addition. The YN sample contained the highest OC, followed by the JL, CQ and JX soil samples, in which the content decreased by 2.04%, 31.10%, and 53.60%, respectively (Fig 1C). The OC content in the YN and JL soil samples was significantly higher than that in the CQ and JX soil samples (*P* < 0.05). In the alkaline soil, the maximum OC content was in the TJ sample, which was 51.64%, 41.43%, and 31.05% significantly higher than those in the IM, GS, and HN soil samples, respectively (Fig 1D). The differences in the OC content in the IM, GS, and HN soil samples were insignificant (*P* > 0.05).

### 3.2. Major soil factors affecting soil OC and accumulative CO_2_ emission

**Fig 2:**
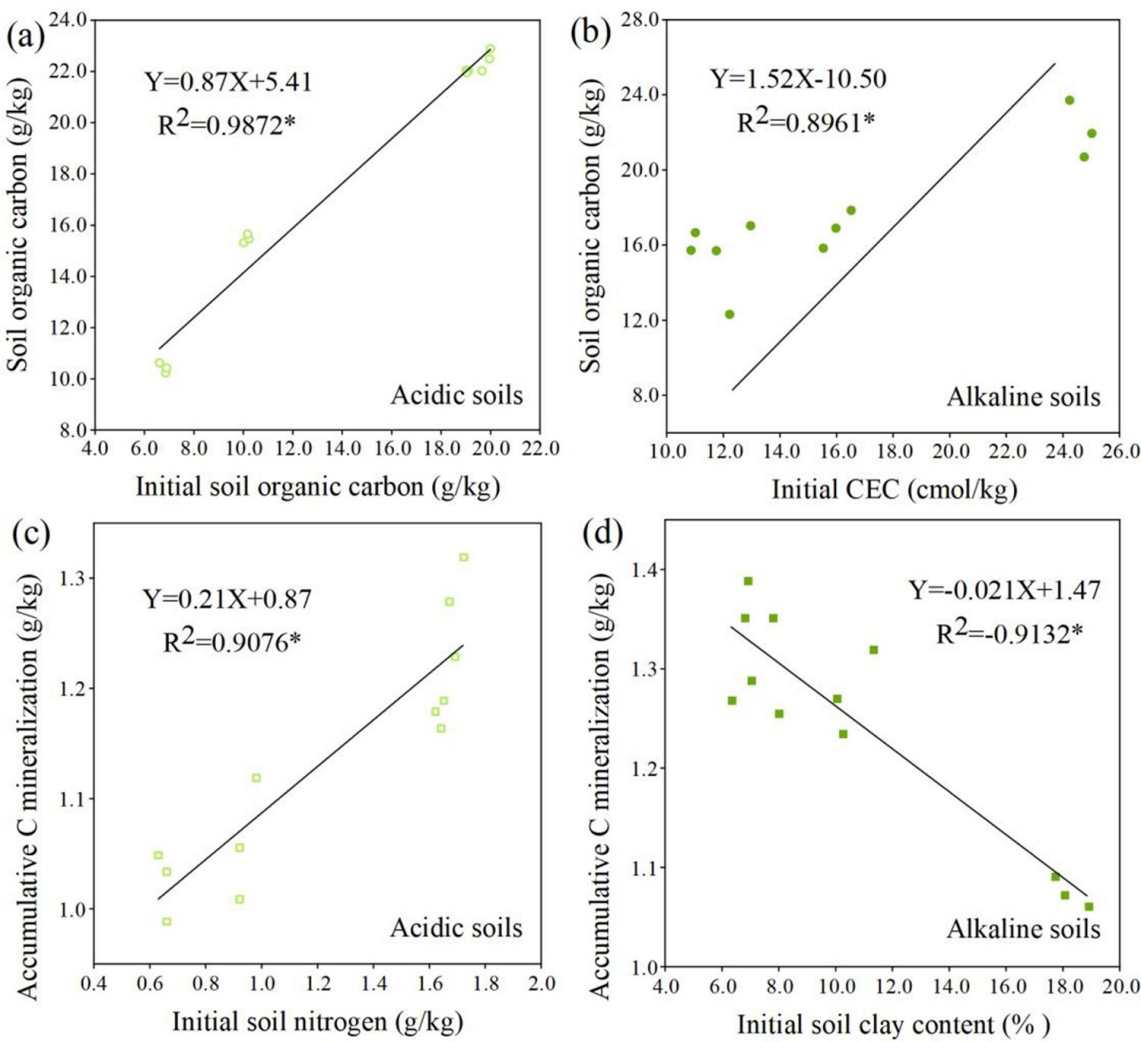
Relationship between initial soil organic carbon and soil organic carbon after the incubation (a) and initial soil nitrogen and accumulative C mineralization after the incubation (c) in the acidic soils, as well as the association between initial CEC content and soil organic carbon after the incubation (b), initial soil clay content and accumulative C mineralization after the incubation (d) in the alkaline soils (Data involved 3 replicates for each indicator; CEC: cation exchange capacity).

Linear regression was introduced to examine the correlation between soil basic properties and soil OC/accumulative C mineralization in different soil types after wheat straw addition. Linear regression analysis was conducted using all eight soil samples separated into acidic and alkaline types. As shown in Fig 2, the soil basic properties with greater correlation coefficients with soil OC/ accumulative C mineralization after incubation were screened. In the acidic soil, there was a highly significant correlation between soil initial OC content and soil OC following incubation (Fig 2A), as well as a significant positive correlation between initial TN content and accumulated C mineralization (Fig 2C). In the alkaline soil, there was a significant relationship between initial CEC and soil OC content (Fig 2B), and a significant negative relationship between soil initial clay content and accumulative C mineralization (Fig 2D).

### 3.3. Carbon-relating bacterial communities and their contributions to OC and accumulative C mineralization

**Fig 3:**
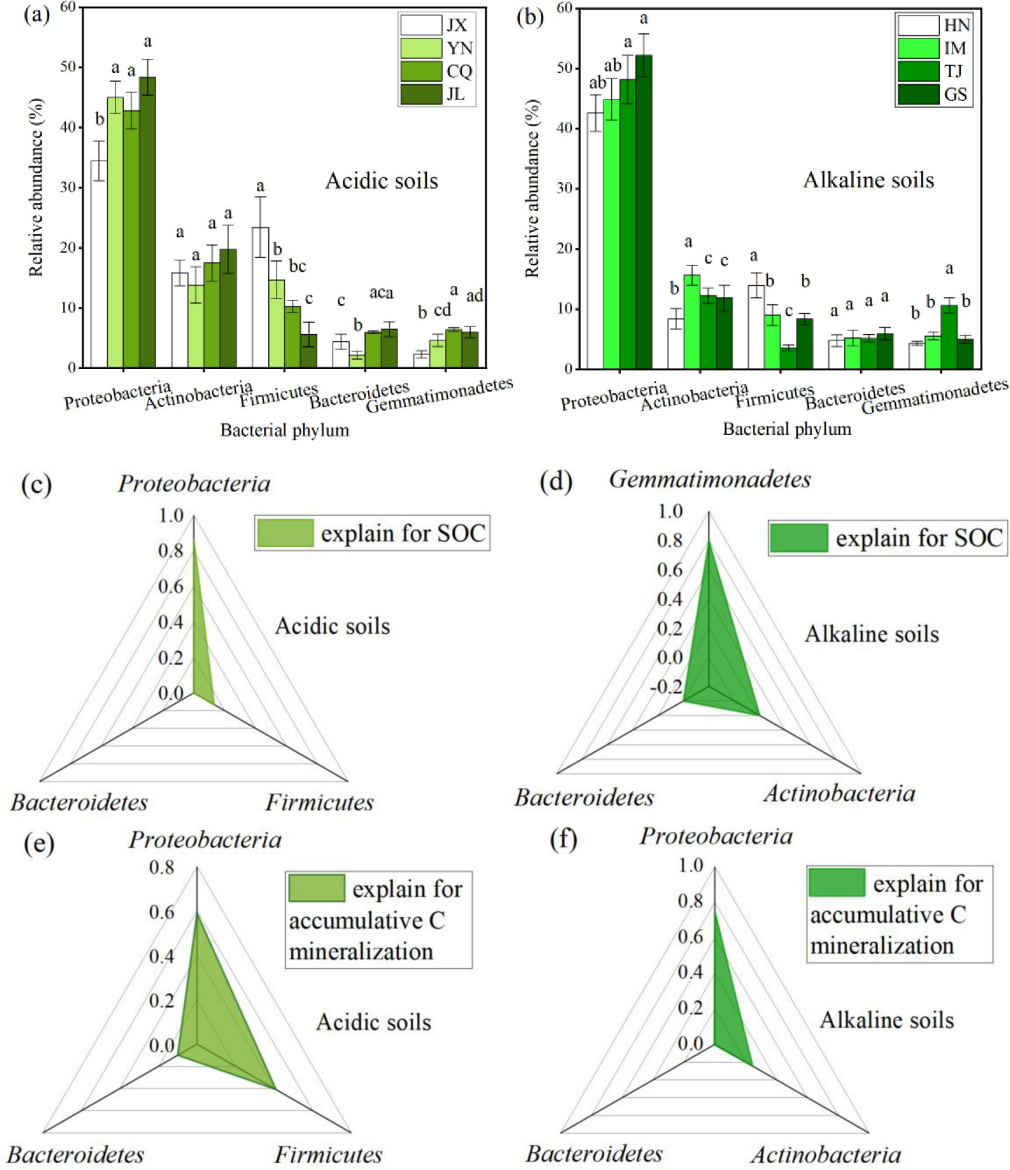
Dominant carbon-relating bacterial phylum after wheat straw addition in the acidic (a) and alkaline soils (b) and their explains for soil organic carbon (c) /accumulative C mineralization (e) in the acidic soils and for soil organic carbon (d) and accumulative C mineralization (f) in the alkaline soils based on the RDA analysis (Values are the mean (n = 3). Different lowercase letters within a column indicate significant differences at the 5% level. Significant difference was performed between that in different types of soils; JX: Jiangxi; JL: Jilin; YN: Yunnan; CQ: Chongqing; GS: Gansu; IM: Inner Mongolia; TJ: Tianjin; HN: Henan).

16S rDNA sequencing was applied to study bacterial dynamics after the incubation. In our study, the operational taxonomic units (OTUs) of 1187–2009 and 2063–2553 were obtained from acidic soils and alkaline soils, respectively. The differences in the number of microorganisms in acid and alkaline soils reflected the significant impact of pH on nutrient transformation after straw returning. Furthermore, carbon-relating bacterial communities at the phylum level were screened from among all dominant bacteria in the soil samples after wheat straw addition (Fig 3). After the incubation, *Proteobacteria*, *Actinobacteria*, *Firmicutes*, *Bacteroidetes*, and *Gemmatimonadetes* were the dominant bacterial phyla in all soil samples and their relative abundances were 73.97% - 86.17%. The dominant bacterial phyla in the acidic and alkaline soils were comparable, differing only in their relative abundance. *Proteobacteria* was the most dominant in soil after wheat straw addition, with a relative abundance of 34.46% - 48.35% in the acidic soil (Fig 3A) and 42.61% - 52.18% in the alkaline soil (Fig 3B), respectively. Furthermore, RDA analysis and explanation were performed to determine the contribution of the dominant phylum to soil OC and accumulative C mineralization in soils. In the acidic soil, the largest explanation for soil OC (Fig 3C) and accumulative C mineralization (Fig 3E) was attributed to *Proteobacteria*, followed by *Firmicutes*. In the alkaline soil, the phylum *Proteobacteria* had the largest explanation for accumulative C mineralization (75.0%), followed by the phylum *Actinobacteria* (24.6%) (Fig 3F); additionally, the phylum *Gemmatimonadetes* accounted for the most soil OC (79.7%), followed by the phylum *Actinobacteria* (20.1%) (Fig 3D).

### 3.4. Soil mineralized nitrogen and TN levels

**Fig 4:**
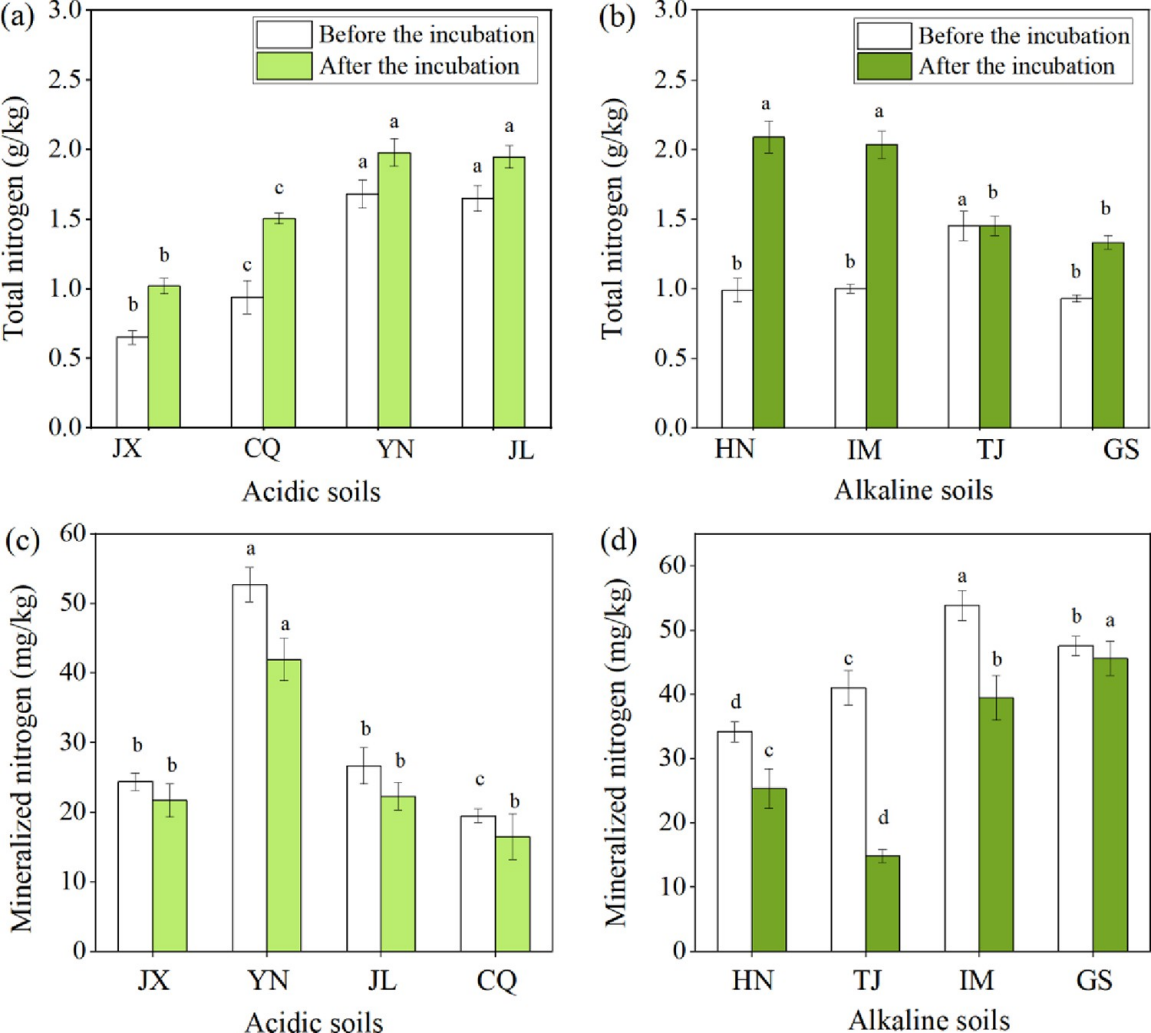
Soil total nitrogen (a) and mineralized nitrogen content (c) in acidic soils and soil total nitrogen (b) and mineralized nitrogen content (d) in the alkaline soils after wheat straw addition (Values are the mean (n = 3). Different lowercase letters within a column indicate significant differences at the 5% level. Significant difference was performed between that in different types of soils; JX: Jiangxi; JL: Jilin; YN: Yunnan; CQ: Chongqing; GS: Gansu; IM: Inner Mongolia; TJ: Tianjin; HN: Henan).

Fig 4 depicts the mineralized nitrogen levels in different soil samples with and without wheat straw addition. Compared with that before incubation, wheat straw addition decreased the mineralized nitrogen levels in all soil types from 4.08% to 63.79%. On day 77 of incubation, mineralized nitrogen levels varied in different soils after wheat straw addition. In the acidic soil, the mineralized nitrogen content was 14.84 mg/kg—45.57 mg/kg in the following sequence: YN, JL, JX, and CQ (Fig 4C). Compared with that in the YN soil sample, the mineralized nitrogen levels significantly decreased by 46.93%, 48.36%, and 60.73% in the JL, JX, and CQ soil samples, respectively. There was no significant difference between the soil mineralized nitrogen levels in JL, JX, and CQ soil samples. In the alkaline soil, GS soil with wheat straw addition had the highest mineralized nitrogen (45.57 mg/kg); this content significantly decreased by 13.48%, 44.39%, and 67.43% in the IM, HN, and TJ soil samples, respectively (Fig 4D). The mineralized nitrogen content in different alkaline soil samples showed a significant difference (*P* < 0.05).

Additionally, wheat straw addition increased TN following incubation. Among the acidic soil, JX soil had the lowest TN content (1.02 g/kg), and its value significantly increased by 47.49%, 90.79%, and 94.02% in CQ, JL, and YN soil samples, respectively (Fig 4A). There was no significant difference between the CQ, JL, and YN soil samples. In the alkaline soil, TN content varied between four soil types, with a range of 1.33 g/kg—2.09 g/kg (Fig 4B). Compared with TN in HN and IM soil samples, its value significantly decreased by 30.48% and 28.63% in TJ and by 36.23% and 34.53% in GS soil samples, respectively.

### 3.5. Major soil factors affecting TN and mineralized nitrogen levels

**Fig 5:**
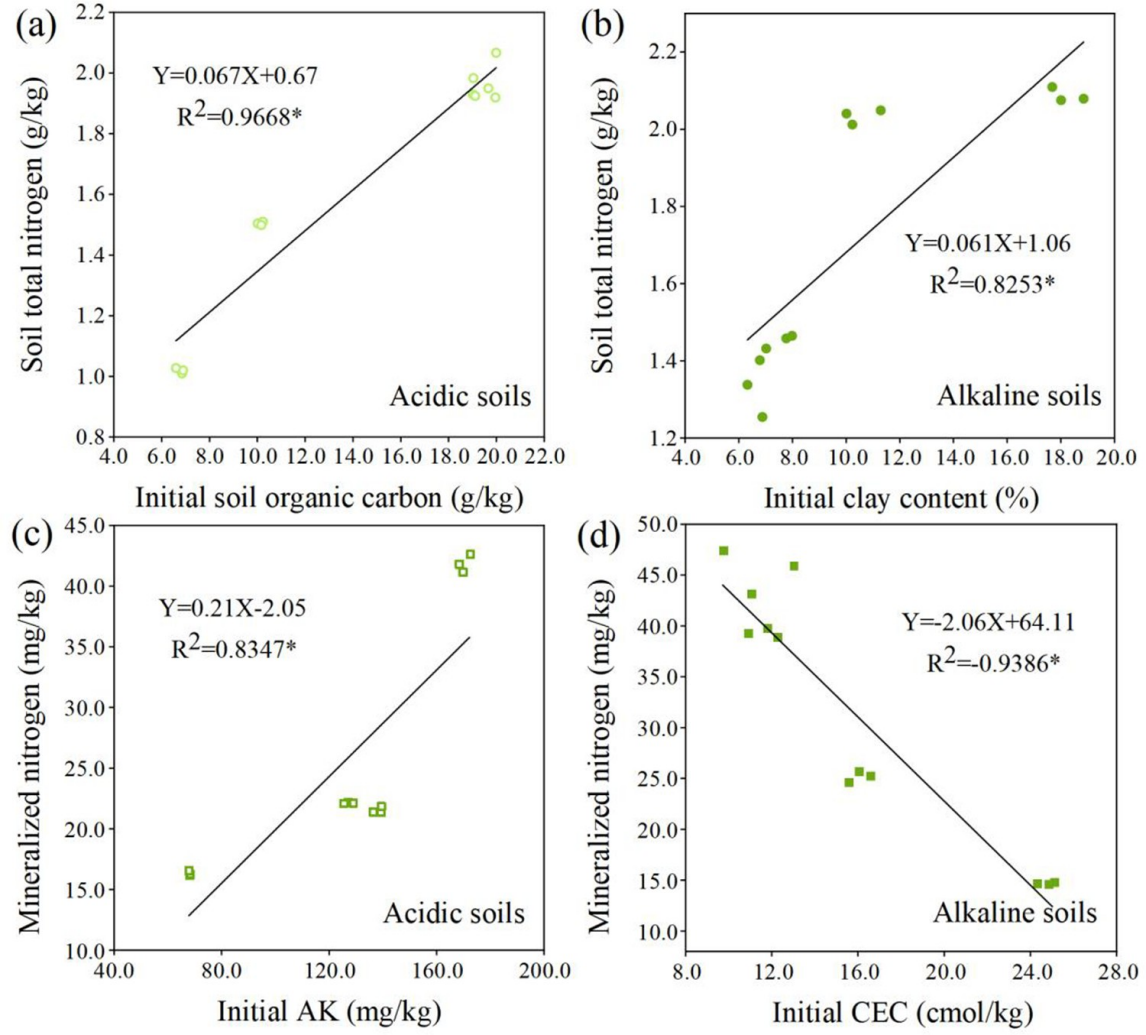
Relationships between initial soil organic carbon and soil total nitrogen after the incubation (a), initial AK content and mineralized nitrogen after the incubation (c) in the acidic soil, as well as the associations between initial clay content and soil total nitrogen after the incubation (b), initial CEC content and mineralized nitrogen after the incubation (d) in the alkaline soil (Data involved 3 replicates for each indicator; AK: available potassium; CEC: cation exchange capacity).

The relationship between soil basic properties and soil TN and mineralized nitrogen levels in the acidic and alkaline soils after wheat straw addition was evaluated by linear regression (Fig 5). The soil basic properties most strongly correlated with soil TN/mineralized nitrogen levels in the acidic and alkaline soils were identified. After incubation, the initial OC content in the acidic soil had the highest correlation coefficient with soil TN (Fig 5A); AK had the highest correlation coefficient with soil mineralized nitrogen levels (Fig 5C). In the alkaline soil, there was a significant correlation between clay content and soil TN after wheat straw addition (Fig 5B) and a significant negative correlation between CEC content and mineralized nitrogen levels (Fig 5D).

### 3.6. Nitrogen-relating bacterial communities and their contributions to soil TN and mineralized nitrogen

**Fig 6:**
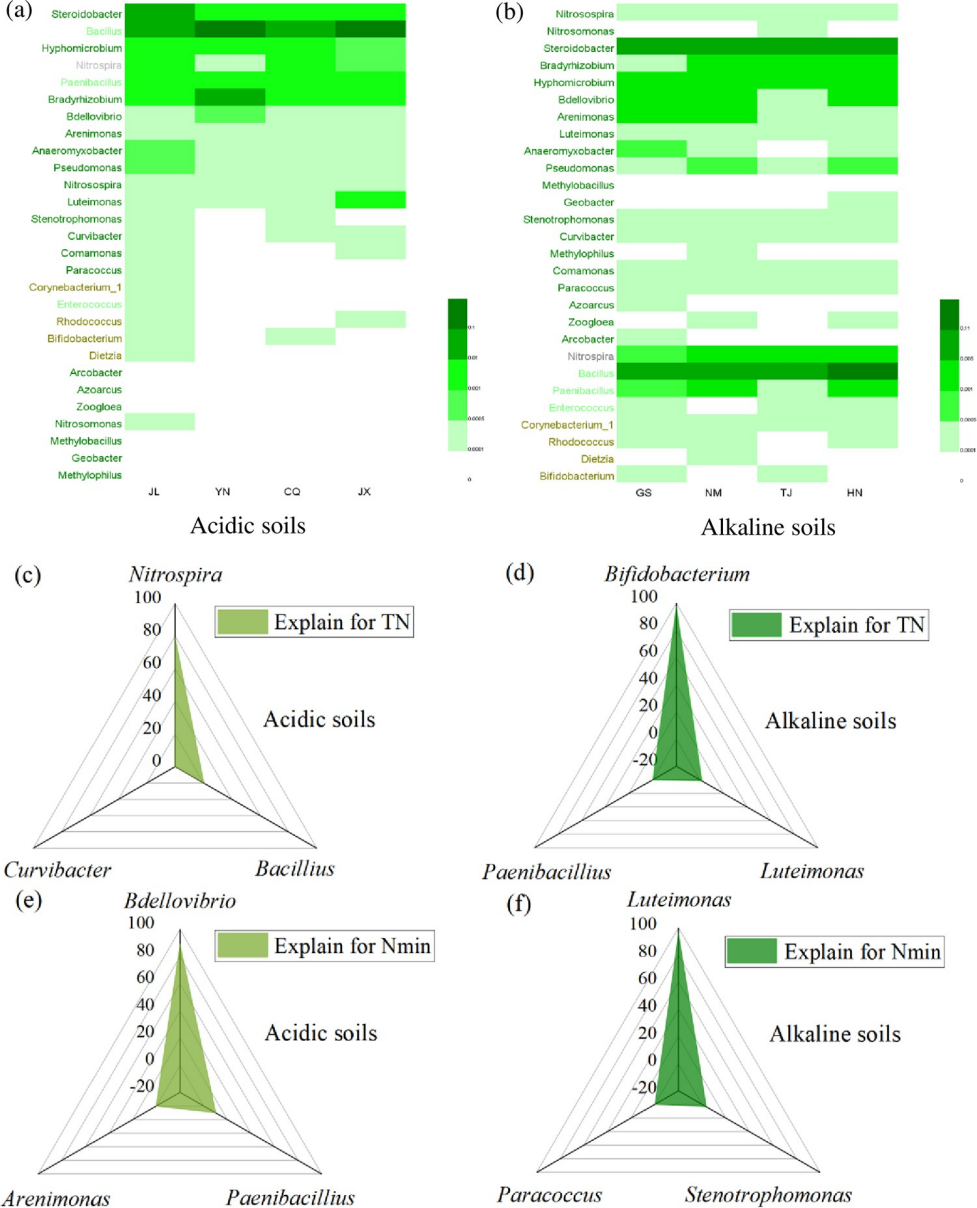
Dominant nitrogen-relating bacterial genera after wheat straw addition in the acidic (a) and alkaline soils (b) and their explains for soil total nitrogen (c) and mineralized nitrogen (e) in the acidic soils and for soil total nitrogen (d) and mineralized nitrogen (f) in the alkaline soils based on the RDA analysis (Values are the mean (n = 3). Different lowercase letters within a column indicate significant differences at the 5% level. Significant difference was performed between that in different types of soils; JX: Jiangxi; JL: Jilin; YN: Yunnan; CQ: Chongqing; GS: Gansu; IM: Inner Mongolia; TJ: Tianjin; HN: Henan).

The dominant nitrogen-relating bacterial genera were identified based on the results obtained from high-throughput sequencing. They belonged to the phyla *Proteobacteria*, *Actinobacteria*, *Firmicutes*, and *Nitrospirae*, which were present in all soil samples after wheat straw addition, with a total relative abundance of 5.26% - 24.01% (Fig 6). The dominant nitrogen-relating bacterial genera differed only in their relative abundance in the acidic and alkaline soils. The genus *Bacillus* was the most dominant nitrogen-relating bacterial genus in the alkaline soil (Fig 6B) and *Steroidobacter* was more abundant in the acidic soil (Fig 6A). RDA analysis and its conditional effects were used to explain the relative contribution of several nitrogen-relating bacterial genera to TN and mineralized nitrogen levels after incubation. For explaining the soil TN after wheat straw addition, *Nitrospira* had the largest explanation in the acidic soil (79.5%) (Fig 6C), whereas *Bifidobacterium* had the largest explanation in the alkaline soil (98.1%) (Fig 6D). However, for the explanation of mineralized nitrogen levels, *Bdellovibrio* better explains its variation in the acidic soil (89.3%) (Fig 6E), whereas *Luteimonas* had the largest explanation rate in the alkaline soil (96.3%) (Fig 6F).

## 4. Discussion

### 4.1. Identification of soil basic properties relating to carbon and nitrogen in the soil

Observing changes in soil OC/TN/mineralized carbon/mineralized nitrogen levels after wheat straw addition contributes to mitigating greenhouse gas effects, improving soil OC, and maintaining soil fertility. The application of organic fertilizer, including wheat straw addition, maintains or enhances soil OC/TN to varying degrees [6, 21, 38]. In our study, soil OC/TN significantly increased in response to the application of wheat straw. Previous studies have indicated that wheat straw addition enhances soil OC/TN by inputting more carbon and nitrogen sources to the soil, providing substrate and energy for microorganisms, and accelerating the production of soil OC and TN [25, 39, 40]. Additionally, wheat straw enhances the capacity of easily mineralized carbon and nitrogen in the soil, whereas wheat straw closely contacts with microbes during mixing with soil, thus increasing soil CO_2_ emission and mineralized nitrogen levels [41]. Furthermore, soil OC/TN/mineralized carbon/mineralized nitrogen levels varied in the acidic and alkaline soils due to variations in their physicochemical properties. Understanding the correlation between soil basic properties and soil OC/TN/mineralized carbon/mineralized nitrogen levels after wheat straw addition is crucial for predicting soil carbon and nitrogen content. The results indicated that in the acidic soil, there was a significant positive correlation between soil basic TN content and accumulative C mineralization, whereas there was a significant positive relationship between soil initial OC and soil OC after incubation. This indicated that the C/N ratio plays a significant role in soil carbon and nitrogen conversion in the acidic soil after straw addition. Soil with a higher OC content promoted carbon and nitrogen fixation by increasing C/N and then accumulated soil carbon and nitrogen after wheat straw returned [42]. Conversely, the higher TN concentration in soil enhanced the decomposition rate of OC/TN [43]. An interesting finding was that the soil initial C/N ratio was significant for soil carbon/nitrogen mineralization and accumulation in the acidic soil than in alkaline soil. Another noteworthy point was that soil with higher initial AK had higher mineralized nitrogen levels after wheat straw returning. In the alkaline soil, clay content positively correlated with TN content after incubation, whereas it had a negative association with accumulative C mineralization. Additionally, CEC positively correlated with soil OC content and negatively correlated with mineralized nitrogen levels. Previous researches have indicated a clear positive correlation between CEC, OM, and pH levels [44, 45]. Furthermore, this verified our hypothesis that higher CEC content causes less OC decomposition, thus accelerating soil OC accumulation. Soil texture affects the combination of amended organic materials with soil particles, as well as the availability of nutrients and microbial substrate [46, 47]. These parameters further influence soil carbon and nitrogen decomposition and enhancement by modifying input and output fluxes of soil OM [48, 49]. Clay-textured soil reduced soil carbon and nitrogen decomposition compared to coarse soil because of the formation of clay-OM stable complexes [50, 51]. For instance, Cˆot´e et al. [51] reported a negative relationship between clay content and OC/TN decomposition in Canadian forest soil. This may be due to the adequate substrate supply, resulting in lower activation energy for microbes in soil with higher clay content [52–54]. However, the results of this study discovered that CEC had a significant relationship with soil nitrogen mineralization than clay content. CEC changes the soil pH, regulating the solubility of OM and its adsorption on a clay surface, thereby influencing OC/TN availability for microbial populations [55]. The adsorption of OM to clay minerals increased with decreasing pH [56]. Therefore, adding clay can increase the adsorption sites in soil, and increasing the CEC content can promote the adsorption of organic materials into clay minerals, enhancing carbon and nitrogen preservation and reducing CO2 emission in the alkaline soil.

### 4.2. Identification of carbon- and nitrogen-relating bacteria in the soil

Straw returning altered the microbial community structures and its dominant microorganisms were identified in previous studies. Yan et al. [57] indicated that *Proteobacteria*, *Acidobacteria*, *Gemmatimonadetes*, *Planctomycetes*, and *Parcubacteria* were dominant in soil after straw returning and positively correlated with soil OC fractions. Cong et al. [58] discovered that wheat straw addition altered the relative abundance of the dominant bacterial phyla *Proteobacteria*, *Acidobacteria*, *Nitrospirae*, and *Verrucomicrobia* in soil. In this study, the phyla *Proteobacteria*, *Actinobacteria*, *Firmicutes*, *Bacteroidetes*, and *Gemmatimonadetes* were identified as the primary microorganisms that decompose soil OC. Variations in the dominant microbial communities may be due to the differences in the experimental setup and materials applied in the soil. The explanation for carbon and nitrogen transformations from RDA analysis revealed that *Proteobacteria* enhanced OC decomposition in the acidic and alkaline soils. The increased abundance of *Proteobacteria* contributed more to carbon storage in the acidic soil, whereas the abundance of *Gemmatimonadetes* explained the increased carbon accumulation in the alkaline soil. *Proteobacteria* dominated both acidic and alkaline soils among all carbon-relating bacteria and played a significant role in carbon transformation [59]. An et al. [60] confirmed that *Gemmatimonadetes* was the dominant bacterial phylum in the alkaline soil. In addition, Yang et al. [61] discovered that the relative abundance of *Gemmatimonadetes* was positively associated with soil MBC concentration. Carbon fixation by microorganisms in the soil confirmed that *Gemmatimonadetes* enhanced the soil OC storage in this study. In terms of microbial community abundance for explaining nitrogen transformations in soil, *Bifidobacterium* explained more for TN and *Luteimonas* explained more for mineralized nitrogen levels in the alkaline soil; whereas *Nitrospira* explained more for TN and *Bdellovibrio* explained more for mineralized nitrogen levels in the acidic soil. In the acidic soil, *Nitrospira* bacteria involved in soil nitrification [62] had a greater influence on TN content. In the alkaline soil, there was a negative correlation between *Bifidobacterium* and TN, as well as an abundance of *Luteimonas* and mineralized nitrogen levels, whereas the positive association between *Nitrospira* and TN, *Bdellovibrio* and mineralized nitrogen levels could be observed in the acidic soil. The findings revealed that regulating the abundance of nitrogen-relating microorganisms can enhance nitrogen sequestration and mineralization in soil.

### 4.3. Recommendations on straw return in production

The correlation between soil basic properties and soil carbon/nitrogen transformations indicated that higher clay content provides more attachment sites for clay-OM complexes. Higher CEC content can promote the absorption of organic materials to clay minerals. In the alkaline soil with higher clay and CEC content, straw return is more beneficial to the accumulation of soil OC and nitrogen. Furthermore, adding clay minerals and salt-based ions to alkaline soil to improve clay and CEC content is an effective approach that ensures carbon/nitrogen storage in soil under straw returns. In the acidic soil with lower C/N ratio, straw returning was more favorable for soil carbon/nitrogen storage. It is worth noting that an increase in AK content in acidic soil can effectively predict the available nitrogen content. Effective replenishment of available potassium in the soil before crop cultivation can enhance the amount of nitrogen available for crop growth under straw returning. Furthermore, increasing the abundance of *Proteobacteria* phylum can enhance carbon transformation in acidic soil, whereas increasing the abundance of *Gemmatimonadetes* phylum can promote carbon sequestration in alkaline soil. Decreased abundance of *Bifidobacterium* and increased abundance of *Luteimonas* can promote nitrogen accumulation in alkaline soil, whereas the increased abundance of *Nitrospira* and decreased abundance of *Bdellovibrio* could promote nitrogen accumulation in acidic soil. Therefore, the addition of clay-mateirals or multiple elements in alkaline soil, as well as the supplementation of AK in acidic soil, plays an important role in regulating carbon and nitrogen conversion after straw returning. Meanwhile, the rational use of relevant bacterial agents can further strengthen the straw management and soil carbon/nitrogen transformation.

## 5. Conclusions

Soil OC/TN/mineralized carbon/mineralized nitrogen varied in different soil types after wheat straw addition. Understanding the relationship between soil basic properties and carbon/nitrogen transformations in different soil types is crucial for identifying its physicochemical and microbial indicators. The findings revealed that in acidic soil with lower C/N, higher soil carbon and nitrogen content could be obtained after wheat straw returning. In the alkaline soil with higher clay and CEC content, wheat straw addition was more effective for soil carbon and nitrogen sequestration. Additionally, regulating microbial abundance can enhance soil carbon and nitrogen transformation. In acidic soil, the phylum *Proteobacteria* can indicate soil carbon transformation, whereas *Nitrospira* and *Bdellovibrio* can indicate soil nitrogen transformation; in the alkaline soil, the phylum *Gemmatimonadetes* can indicate soil carbon sequestration, whereas *Bifidobacterium* and *Luteimonas* can indicate soil nitrogen transformation. These soil physicochemical and biological indicators need to be further validated to provide scientific recommendations on soil carbon and nitrogen sequestration by straw returning to different types of soil.

## Supporting information

S1 Table The basic properties of soils before the incubation. *CEC: cation exchange capacity; TOC: total organic carbon; TN: total nitrogen; AP: available phosphorus; AK: available potassium.
(DOCX)
