# Supplementary material for: Identification of the soil physicochemical and bacterial indicators for soil organic carbon and nitrogen transformation under the wheat straw returning
Source: PLoS One. 2024 Apr 4;19(4):e0299054. doi: 10.1371/journal.pone.0299054 (PMC10994409; doi:10.1371/journal.pone.0299054)
Supplement: S1 Table — *CEC: cation exchange capacity; TOC: total organic carbon; TN: total nitrogen; AP: available phosphorus; AK: available potassium. (DOCX) [file pone.0299054.s001.docx]

**Supporting Information**

**Identification of the soil physicochemical and bacterial indicators for**

**soil organic carbon and nitrogen transformation**

**under the wheat straw returning**

Yajun Yang^a,^*, Hui Wang^b^, Chengjuan Li^b^, Hexiang Liu^a^, Xianhui Fang^a^, Mengyuan Wu^a^, Jialong Lv^a,^*

^a^*State Key Laboratory of Soil Erosion and Dryland Farming on the Loess Plateau, Institute of soil and water conservation Chinese Academy of Sciences & College of Natural Resources and Environment, Northwest A&F University, Yangling, Shaanxi Province 712100, PR China*

*^b^Xianyang Soil and Fertilizer Workstation, Xianyang, Shaanxi Province 712000, PR China*

1. **Materials and methods**

*2.2. Determination methods*

**S1 Table** The basic properties of soils before the incubation

| Soils | Locations | Properties | | | | | | | | |
| --- | --- | --- | --- | --- | --- | --- | --- | --- | --- | --- |
|  |  | Clay (%) | CEC (cmol·kg^-1^) | pH | CaCO_3_ (g·kg^-1^) | TOC (g·kg^-1^) | TN (g·kg^-1^) | AP  (mg·kg^-1^) | AK  (mg·kg^-1^) | Background Cd (mg·kg^-1^) |
| Inner Mongolia | 41°33' N, 110°01' E | 10.51 | 11.61 | 8.80 | 11.51 | 9.45 | 1.00 | 13.89 | 119.6 | 0.22 |
| Gansu | 38°52' N, 100°26' E | 6.66 | 11.23 | 8.37 | 38.51 | 11.18 | 0.93 | 26.30 | 135.7 | 0.21 |
| Henan | 35°00' N, 113°41' E | 18.18 | 16.01 | 8.07 | 27.50 | 10.32 | 0.99 | 60.00 | 79.15 | 0.23 |
| Tianjin | 38°45' N, 117°06' E | 7.59 | 24.67 | 8.29 | 53.57 | 12.77 | 1.45 | 59.91 | 210.3 | 0.22 |
| Chongqing | 29°48' N, 106°24' E | 24.96 | 21.34 | 5.74 | 0.00 | 10.14 | 0.94 | 46.60 | 68.05 | 0.20 |
| Jilin | 43°31' N, 124°48' E | 30.18 | 31.11 | 6.82 | 0.00 | 19.05 | 1.65 | 82.88 | 127.0 | 0.14 |
| Jiangxi | 28°12' N, 116°56' E | 36.51 | 8.70 | 6.01 | 0.00 | 6.78 | 0.65 | 30.34 | 138.2 | 0.18 |
| Yunnan | 24°52' N, 102°49' E | 27.52 | 11.10 | 5.92 | 0.00 | 19.87 | 1.68 | 54.53 | 170.0 | 0.30 |
